# Strain-level profiling of viable microbial community by selective single-cell genome sequencing

**DOI:** 10.1038/s41598-022-08401-y

**Published:** 2022-03-15

**Authors:** Masahito Hosokawa, Taruho Endoh, Kazuma Kamata, Koji Arikawa, Yohei Nishikawa, Masato Kogawa, Tatsuya Saeki, Takuya Yoda, Haruko Takeyama

**Affiliations:** 1 Department of Life Science and Medical Bioscience, Waseda University, 2-2 Wakamatsu-cho, Shinjuku-ku, Tokyo, 162-8480 Japan; 2 Research Organization for Nano and Life Innovation, Waseda University, 513 Wasedatsurumaki-cho, Shinjuku-ku, Tokyo, 162-0041 Japan; 3 bitBiome, Inc., 513 Wasedatsurumaki-cho, Shinjuku-ku, Tokyo, 162-0041 Japan; 4 Computational Bio Big-Data Open Innovation Laboratory, National Institute of Advanced Industrial Science and Technology, 3-4-1 Okubo, Shinjuku-ku, Tokyo, 169-8555 Japan; 5 Institute for Advanced Research of Biosystem Dynamics, Waseda Research Institute for Science and Engineering, 3-4-1 Okubo, Shinjuku-ku, Tokyo, 169-8555 Japan

**Keywords:** Metagenomics, Microbiome, Bacteria, Microbiology techniques, DNA sequencing, Whole genome amplification

## Abstract

Culture-independent analysis with high-throughput sequencing has been widely used to characterize bacterial communities. However, signals derived from non-viable bacteria and non-cell DNA may inhibit its characterization. Here, we present a method for viable bacteria-targeted single-cell genome sequencing, called PMA-SAG-gel, to obtain comprehensive whole-genome sequences of surviving uncultured bacteria from microbial communities. PMA-SAG-gel uses gel matrixes that enable sequential enzymatic reactions for cell lysis and genome amplification of viable single cells from the microbial communities. PMA-SAG-gel removed the single-amplified genomes (SAGs) derived from dead bacteria and enabled selective sequencing of viable bacteria in the model samples of *Escherichia coli* and *Bacillus subtilis*. Next, we demonstrated the recovery of near-complete SAGs of eight oxygen-tolerant bacteria, including *Bacteroides* spp. and *Phocaeicola* spp., from 1331 human feces SAGs. We found the presence of two different strains in each species and identified their specific genes to investigate the metabolic functions. The survival profile of an entire population at the strain level will provide the information for understanding the characteristics of the surviving bacteria under the specific environments or sample processing and insights for quality assessment of live bacterial products or fecal microbiota transplantation and for understanding the effect of antimicrobial treatments.

## Introduction

Culture-independent DNA-based analysis has been widely used to characterize bacterial communities in various microbiome analyses. Next-generation sequencer (NGS)-based high-throughput sequencing analyzes total DNA in samples containing viable bacteria, non-viable bacteria, and cell-free DNA. However, signals derived from non-viable cell DNA may impede the characterization of viable microbes in the total bacterial communities^1^. For example, it has been reported that non-viable cell DNA may alter the results of assays carried out for evaluating the impact of viable bacteria in fecal microbiota transplantation (FMT) protocols^2–4^ or the efficacy of antimicrobial compounds in the sputum specimens of cystic fibrosis patients^5,6^. Thus, discerning viable from non-viable bacteria in these assays is of major importance. Since there is no method that can determine the survival profile of an entire population at the species or strain level, a comprehensive method for the analysis of genomic information from surviving non-cultured bacteria is necessary.

One approach for characterizing viable bacteria from the microbial community is an assay combined with a chemical treatment for the removal of amplification signals derived from extracellular or non-viable cell DNA. The most commonly used chemical agent for this purpose is propidium monoazide (PMA)^7^. PMA is a cell membrane-impermeable DNA-modifying dye that is photoactivated and covalently binds to dsDNA, preventing polymerase-based DNA amplification^8^. Thus, only DNA from viable cells with intact cell membranes will be amplified and detected as a signal from analyzed microbial communities. This treatment has been used in combination with the polymerase chain reaction (PCR)^9,10^, amplicon (16S rRNA gene) sequencing^2–4,11^, and shotgun metagenomics^12,13^ to selectively detect viable bacteria in various microbial communities and to determine the relationship between the diversity of viable bacteria and environmental conditions.

In combination with PMA treatment, metagenomics approaches, including 16S rRNA gene amplicon sequencing and shotgun metagenomic sequencing, can provide comprehensive insight into microbial composition and function of viable bacterial cell populations. However, while 16S rRNA gene sequencing provides snapshots of microbial composition, it does not address bacterial metabolic function^14^. Similarly, shotgun metagenomics allows for the comprehensive study of the genomic content of microbial communities, but linking taxonomic and functional information for individual bacterial strains remains a challenge^15,16^. These analyses rely exclusively on the mapping identification of sequence reads, and thus the accuracy of analysis depends on the availability of reference bacterial genomes for accurate taxonomic and functional assignment^17,18^. However, reference genomes are still lacking even for the most well-studied microbiome, that of humans. Therefore, an ideal approach for the classification and functional characterization of uncultured viable bacteria would be to obtain their whole-genome sequences by specifically targeting viable bacteria from complex microbial communities.

In this study, we developed a method that combines single-cell genome sequencing with PMA treatment to obtain comprehensive whole-genome sequences of viable bacteria from microbial communities. A massively parallel single bacterial genome sequencing technology, called PMA-SAG-gel, uses gel matrices that enable sequential enzymatic reactions for cell lysis and genome amplification of single cells from PMA-treated microbial communities^19^. We validated the technology using model samples of *Escherichia coli* and *Bacillus subtilis*. Further, we demonstrated the recovery of high-quality single-amplified genomes (SAGs) of oxygen-tolerant *Bacteroides* spp. and *Phocaeicola* spp. from PMA-treated human feces. The nearly complete genomes obtained through viable single-cell genome sequencing provide genomic information that enables the functional strain-level characterization of viable cells, which was not feasible via previous PMA assays. Viable bacterial genomes obtained through PMA-SAG-gel can be used for the identification of metabolically active bacteria from microbial communities, regardless of cultivability, and may provide information for the quality assessment of live bacterial products and their appropriate processing.

## Methods

### Experimental design and fecal sample collection

The human subjects have all signed a written informed consent and the project has been approved by the ethics review committee at Waseda University (No. 2018-323). All methods used were conducted in accordance with the guidelines and regulations outlined by the ethics approval. Fresh feces (WSD032 and WSD033) were collected by subjects in 15 mL vials and stored on ice for a maximum of 3 h. Preserved feces were also collected in 15 mL vials containing 3 mL GuSCN solution (TechnoSuruga Laboratory Co., Ltd.) and stored for a maximum of 2 days prior to single-cell encapsulation in droplets.

Following homogenization of human feces in PBS or GuSCN solution (500 μL), the supernatant was recovered by centrifugation at 2000×*g* for 30 s, followed by filtration through a 35-μm nylon mesh and centrifugation at 8000×*g* for 5 min. The resulting cell pellets were suspended in PBS and centrifuged twice at 8000×*g* for 5 min.

### Preparation of model bacteria suspensions

For genome sequencing analysis, *E. coli* K-12 strain (ATCC 10798; genome size: 4.6 Mbp) and *B. subtilis* (ATCC 6633; genome size: 4.0 Mbp) were used as model bacteria. *E. coli* K-12 cells were pre-cultured in Luria–Bertani medium for 16 h. *B. subtilis* cells were pre-cultured in brain heart infusion broth for 16 h. For cell collection, 1 mL of culture medium was dispensed into a 1.5 mL tube and centrifuged at 8000×*g* for 5 min. After removing the supernatant, the collected cells were resuspended in UV-treated Dulbecco’s phosphate-buffered saline (DPBS, Thermo Fisher Scientific) and washed three times with DPBS. Finally, the cells were resuspended in 500 μL DPBS, and the cell concentration was calculated using a bacterial counter. All preparations for the cell suspension and further processes were performed under an open-interior clean bench KOACH T 500-F (KOKEN LTD.) or a biosafety cabinet (Esco Micro Pte. Ltd.), except for droplet generation and isolation with fluorescence-activated cell sorting (FACS).

### Viable single-cell genome sequencing with PMA-SAG-gel

150 μL of bacterial suspension prepared to 1.0 × 10^6^ cells/μL was taken in a 1.5 mL tube. In heat-treated samples, the suspension was treated at 95 °C for 5 min or 85 °C for 10 min. PMA (Biotium) was then added to the bacterial suspension to a final concentration of 1, 5, or 12.5 μM. After 5 min of incubation at room temperature, the suspension was illuminated with an LED Crosslinker 12 (Takara Bio Inc.) for 15 min. The pellet was then centrifuged at 8000×*g* for 5 min and washed twice with PBS.

Single-cell genome amplification was performed using the SAG-gel platform, as described in our previous reports^19^. Prior to single-cell encapsulation, cell suspensions were adjusted to 0.3–0.4 cells/droplets in 1.5% agarose in PBS to prevent encapsulation of multiple cells in single droplets. Using an On-chip Droplet Generator (On-chip Biotechnologies Co., Ltd.), single bacterial cells were encapsulated in droplets and collected in a 1.5 mL tube, which was chilled on ice for 15 min to form the gel matrix. Following solidification, the collected droplets were broken with 1H, 1H, 2H, 2H-perfluoro-1-octanol (Sigma-Aldrich) to collect the capsules. The gel capsules were washed with 500 μL acetone (FUJIFILM Wako Pure Chemical Corporation), and the solution was mixed vigorously and centrifuged. The acetone supernatant was removed, 500 μL isopropanol (FUJIFILM Wako Pure Chemical Corporation) was added, and the solution was once again mixed vigorously and centrifuged. The isopropanol supernatant was then removed, and the gel capsules were washed three times with 500 μL DPBS.

Individual cells in capsules were then lysed by submerging the gel capsules in lysis solutions: first, 50 U/μL Ready-Lyse Lysozyme Solution (Lucigen), 2 U/mL Zymolyase (Zymo Research Corporation), 22 U/mL lysostaphin (Sigma-Aldrich), and 250 U/mL mutanolysin (Sigma-Aldrich) in DPBS at 37 °C overnight; second, 0.5 mg/mL achromopeptidase (FUJIFILM Wako Pure Chemical Corporation) in PBS at 37 °C for 6–8 h; and third, 1 mg/mL Proteinase K (Promega Corporation) with 0.5% SDS in PBS at 40 °C overnight. At each reagent replacement step, the gel capsules were washed three times with DPBS and subsequently resuspended in the next solution. Following lysis, the gel capsules were washed with DPBS five times, and the supernatant was removed. The capsules were then suspended in Buffer D2 and subjected to multiple displacement amplification (MDA) using a REPLI-g Single Cell Kit (QIAGEN). Following MDA at 30 °C for 3 h, the gel capsules were washed three times with 500 μL of DPBS. Thereafter, the capsules were stained with 1 × SYBR Green I (Thermo Fisher Scientific) in DPBS and observed with fluorescence microscopy BZ-X810 (KEYENCE CORPORATION) to count the number of fluorescence-positive gel capsules. The number of drops was counted using ImageJ software (National Institutes of Health). Following confirmation of DNA amplification based on the presence of green fluorescence in the gel, fluorescence-positive capsules were sorted into 384-well plates using a BD FACSMelody cell sorter (BD Bioscience) equipped with a 488-nm excitation laser. Following droplet sorting, 384-well plates proceeded to the second round of MDA or were stored at − 30 °C. Following gel capsule collection in 384-well plates, second-round MDA was performed using the REPLI-g Single Cell Kit. Buffer D2 (0.3 μL) was added to each well and incubated at 65 °C for 10 min. Thereafter, 1.7 μL of MDA mixture was added and incubated at 30 °C for 120 min. The MDA reaction was terminated by heating at 65 °C for 3 min.

### Single-cell sequencing

For sequencing analysis, sequencing SAG libraries were prepared from the second-round MDA product using QIAseq FX DNA Library Kit (QIAGEN). Aliquots of SAGs were transferred to replica plates for DNA yield quantification using Quant-iT dsDNA Broad-Range (BR) Assay Kit (Thermo Fisher Scientific) prior to library preparation. Ligation adaptors were modified to TruSeq-Compatible Full-length Adapters UDI (Integrated DNA Technologies, Inc.). Each SAG library was sequenced using MiSeq System 2 × 75 bp configuration or NextSeq 2000 System 2 × 150 bp configuration (Illumina, Inc.).

### qPCR analysis

The reaction mix was prepared according to the manual of KAPA SYBR Fast qPCR kit (Kapa Biosystems) using the *E. coli*-specific primer^20^ and measured using StepOnePlus Real-Time PCR System (Thermo Fisher Scientific).

### SAG analysis

Sequence reads were processed to eliminate low-quality reads using fastp 0.20.1^21^ with default options or BBDuk 38.79^22^ (options: qtrim = r, trimq = 10, minlength = 40, maxns = 1, minavgquality = 15, ktrim = r ref = adapters k = 23 mink = 11 hdist = 1 tpe tbo). Human genome contamination was removed from the sequence reads by mapping to reference human genome with BBMap 38.79 (options: quickmatch fast untrim minid = 0.95, maxindel = 3, bwr = 0.16, bw = 12, minhits = 2, path = human_masked_index(*), qtrim = rl, trimq = 10). Short reads were assembled de novo using SPAdes 3.14.0 (options for SAG: -sc -careful -disable-rr -disable-gzip-output -t 4 -m 32), and contigs < 1000 bp were excluded from subsequent analyses^23^.

### Grouping of the same strain SAGs into CoSAG

SAGs with completeness > 20% and contamination of < 10% were selected using CheckM lineage workflow^24^. The average nucleotide identity (ANI) was calculated for the selected SAGs using FastANI 1.3^25^. SAGs with ANI > 98%, single-copy marker gene homology > 99.9%, and tetra-nucleotide frequency correlation > 90% were identified in the same strain group with hierarchical clustering. From each group of strains, 3–8 SAGs with a total contamination rate of less than 5% were selected and co-assembled. Sequence reads from one SAG were mapped to other SAGs in the same group using MINIMAP2 2.17^26^ (options: -ax sr). According to the ccSAG procedure^27^, the partially aligned potential chimeras were split into aligned and unaligned fragments. Short fragments (< 20 bp) were discarded. Clean and chimera-removed reads were obtained using cycles of cross-reference mapping and chimera splitting for each sample in the same group. Quality-controlled reads from the same group were co-assembled de novo as CoSAG using SPAdes (options: -sc -careful -disable-rr -disable-gzip-output -t 4 -m 32).

### Gene prediction, taxonomy identification, and plasmid detection

CDS, rRNAs, and tRNAs were extracted from all SAGs using Prokka 1.14.6^28^ (option: rawproduct -mincontiglen 200). 16S and 23S rRNA genes with lengths of ≥ 700 and 1100 bp, respectively, were detected. Taxonomy identification was performed using GTDB-Tk 1.3.0^29^ with the default option and the Release95 database. Anvi’o was used to compare the strain genomes to extract core and accessory genes with COG annotations^30^. KEGG Decoder v 1.0.8.2 was used to determine the completeness of various metabolic pathways based on a set of key genes^31^.

### Ethics approval and consent to participate

The human subjects have all signed a written informed consent and the project has been approved by the ethics review committee at Waseda University (No. 2018-323). All methods used were conducted in accordance with the guidelines and regulations outlined by the ethics approval.

## Results

### Evaluation of the inhibition of genome amplification of non-viable single cells

**Figure 1:**
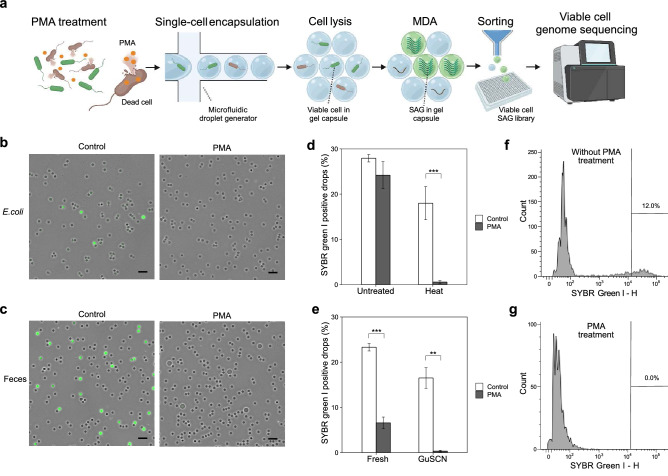
Viable bacteria-targeted single-cell genome amplification with PMA-SAG-gel. (**a**) Workflow of PMA-SAG-gel for single-cell genome sequencing of viable bacterial cells. The bacterial suspension was treated with PMA to prevent DNA amplification from the dead cells. The cells were then randomly captured in gel capsules via a microfluidic droplet generator and processed via in-gel cell lysis and multiple displacement amplification (MDA) in a test tube. Single-amplified genomes (SAGs) obtained from viable cells were fluorescently detected in the gel and sorted into well plates as SAG library for DNA sequencing. (**b**, **c**) Fluorescent images of gel capsules stained with SYBR green I after MDA from heat-killed *E. coli* (**b**) and feces suspended in guanidine thiocyanate (GuSCN) solution (**c**). Green signals indicate amplified genomes in gel capsules. Control indicates no PMA treatment. Scale bar; 100 μm. (**d**, **e**) Number of gel capsules stained with SYBR green I after MDA from untreated or heat-killed *E. coli* (**d**) and fresh feces or feces suspended in GuSCN solution (**e**). Data are presented as the mean ± SD (n = 3). **p < 0.01; ***p < 0.005 (Student’s *t-test*). (**f**, **g**) Fluorescent intensity histogram of gel capsules analyzed on a flow cytometer using feces suspended in GuSCN without (**f**) or with (**g**) PMA treatment. The gates show the gel capsules to be sorted for subsequent sequencing analysis.

First, we evaluated the applicability of PMA for the inhibition of whole-genome amplification from non-viable bacterial single cells. Heat-treated *E. coli* or feces suspended in guanidine thiocyanate (GuSCN) solution were used as model samples. The GuSCN solution is frequently used for storing feces at room temperature^32^. Bacterial suspensions were prepared from each sample and pretreated with PMA. The bacterial suspension was then introduced into the microfluidic channel to generate single-cell-captured gel capsules through the SAG-gel method^19^ (Fig. 1a).

As shown for PMA *E. coli* in Fig. 1b, when the single-cell genome was amplified via the in-gel MDA reaction, fluorescent signals were observed in the gel capsules after staining with DNA-intercalating dye. We observed that the fluorescence signal was significantly attenuated in the gel capsule, which captured PMA-treated heat-killed *E. coli* (PMA-heat *E. coli*). In the PMA-treated fecal samples, we found a certain number of fluorescence-positive capsules among PMA-fresh feces, while the fluorescence signal was significantly attenuated in PMA-GuSCN feces (Fig. 1c). The ratios of fluorescence-positive capsules in the PMA-treated non-viable bacterial samples decreased significantly to less than 0.6% in PMA-heat *E. coli* (18.0% in non-PMA) and 0.3% in PMA-GuSCN feces (16.5% in non-PMA), respectively (Fig. 1d,e), suggesting that genome amplification was effectively suppressed via PMA treatment. In contrast, in the non-killed samples (PBS-*E. coli* and fresh feces), the positive capsule fractions decreased through PMA treatment (27.9% to 24.2% in viable *E. coli* and 23.3% to 6.59% in fresh feces) as compared to non-PMA treatment, but this was considered a reasonable variation based on the native cell viabilities.

In the SAG-gel process, fluorescence-positive gel capsules were collected via FACS. Among viable bacteria (*E. coli*), the proportion of fluorescence-positive gel capsules to be sorted was 4.1% without PMA treatment and 6.2% with PMA treatment, which was within the range predicted by the cell ratio variation in the PMA treatment process, indicating that PMA did not interfere with the sorting of viable bacteria. In the case of dead bacteria (heat-treated *E. coli* and GuSCN-treated feces), the ratios of gel capsules to be sorted were 1.1% and 12.0%, respectively, without PMA treatment, and both were 0% after PMA treatment (Fig. 1f,g), suggesting that the DNA from dead bacteria was not transferred and could be effectively eliminated via the gel capsule sorting process.

### Effect of PMA treatment on single-cell whole-genome sequencing

**Figure 2:**
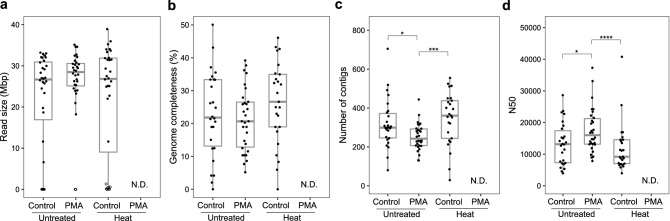
Effect of PMA treatment on single-cell whole-genome sequencing. Untreated and heat-killed *E. coli* samples were reacted with PMA and processed with SAG-gel for whole-genome sequencing. (**a**) Size of raw sequence reads. An SAG < 1 Mb is indicated by a white circle. (**b**) Genome completeness of SAGs. (**c**) Number of contigs in the SAGs. (**d**) N50 of SAGs. 32 SAGs were sequenced for each sample. In (**b–d**), SAGs with < 1 Mb reads were removed. PMA-heat-*E. coli* showed no fluorescent gel capsules, so we could not determine their sequencing states (N.D. not determined). Control means no PMA treatment. *p < 0.05; ***p < 0.005; ****p < 0.001 (Wilcoxon rank-sum test).

Next, we performed whole-genome sequencing of the sorted single *E. coli* gel capsules to evaluate the effect of PMA treatment on sequencing quality. First, PMA-heat *E. coli* was excluded from the evaluation because genome amplification was considerably suppressed, and gel capsule sorting could not be performed (Fig. 2).

The average sequence read sizes were not significantly different (21–27 Mb) among the non-PMA-*E. coli*, PMA-*E. coli*, and non-PMA-heat *E. coli* (Fig. 2a). It is worth noting that some sequencing results with extremely small sequence reads were present in the non-PMA samples to a certain degree (19% in non-PMA-*E. coli* and 22% in non-PMA-heat *E. coli*), but decreased to 6.3% in PMA-*E. coli*. These low-output sequence reads were consistent with the samples that had insufficient DNA yield in the second MDA after gel capsules sorting. This reduction in inadequate DNA amplification could come as a result of PMA eliminating DNA amplification from non-viable bacteria and free DNA.

Regarding the quality of de novo assembled genomes, the average genome completeness was about 20% (Fig. 2b), and there was no significant difference with regard to PMA treatment. The genome completeness of 20% was a reasonable value because the sequencing reads were obtained with coverages of approximately six times the *E. coli* genome (4.6 Mbp). In contrast, PMA-*E. coli* exhibited a significant decrease in contigs (320 in non-PMA to 252 in PMA) (Fig. 2c) and an increase in N50 (13.3 kb in non-PMA to 17.5 kb in PMA) (Fig. 2d) relative to non-PMA-*E.coli*. This quality improvement was considered the result of fragmented DNA molecules in non-viable cells being eliminated via PMA treatment. Single-cell genome amplification from near-intact DNA from viable cells could therefore be achieved. Thus, PMA treatment was expected to improve the quality of single-cell genomes, even from clonal samples, by eliminating products derived from dead bacteria.

### Elimination of dead bacteria through PMA treatment and selective sequencing of viable bacteria

**Figure 3:**
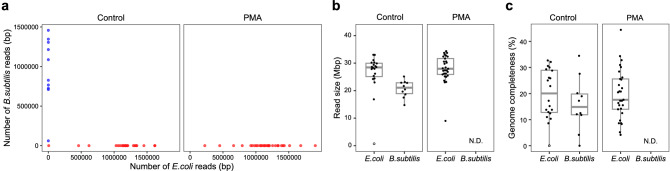
Verification of viable bacterial selectivity of single-cell sequencing via PMA-SAG-gel. PMA-SAG-gel sequenced a mixture of *E. coli* and heat-killed *B. subtilis* cells. (**a**) scatter plot shows the number of reads mapped to *E. coli* and *B. subtilis* genomes associated with each SAG (capsule). Red dots indicate droplets that were identified as *E. coli* from 16S rRNA data; blue dots indicate droplets that were identified as *B. subtilis*. (**b**) Size of raw sequence reads. (**c**) Genome completeness of SAGs. 32 SAGs were sequenced for each sample. PMA-heat-*B. subtilis* showed no fluorescent gel capsules, so we could not determine their sequencing states (N.D. not determined).

To prove that our method can yield viable bacteria-specific single-cell genomic information, we performed PMA-SAG-gel sequencing using a mixture of equal parts viable *E. coli* and heat-treated *B. subtilis*. After sorting the fluorescent-positive gel capsules, 32 SAGs (capsules) were randomly sequenced under non-PMA or PMA treatment conditions. Figure 3 presents a plot of the number of SAG reads mapped to the *E. coli* and *B. subtilis* genomes. All non-PMA SAG reads were exclusively mapped to *E. coli* or *B. subtilis*, indicating no sequence contamination between different species (Fig. 3a). However, the PMA-SAG sequence reads that were dominantly mapped to *B. subtilis* disappeared, indicating that only *E. coli* SAGs were obtained under these conditions (Fig. 3a). In addition, the genome quality of the mixed sample was the same as that of *E. coli* alone samples, and genome completeness was maintained. Since the sequencing results were obtained with a sixfold coverage, the 20% completeness was reasonable (Fig. 3b,c). These results indicated that PMA-SAG-gel could remove the SAGs derived from dead bacteria and enable the selective sequencing of viable bacteria.

### Evaluation of the sequence of viable bacteria from feces

**Figure 4:**
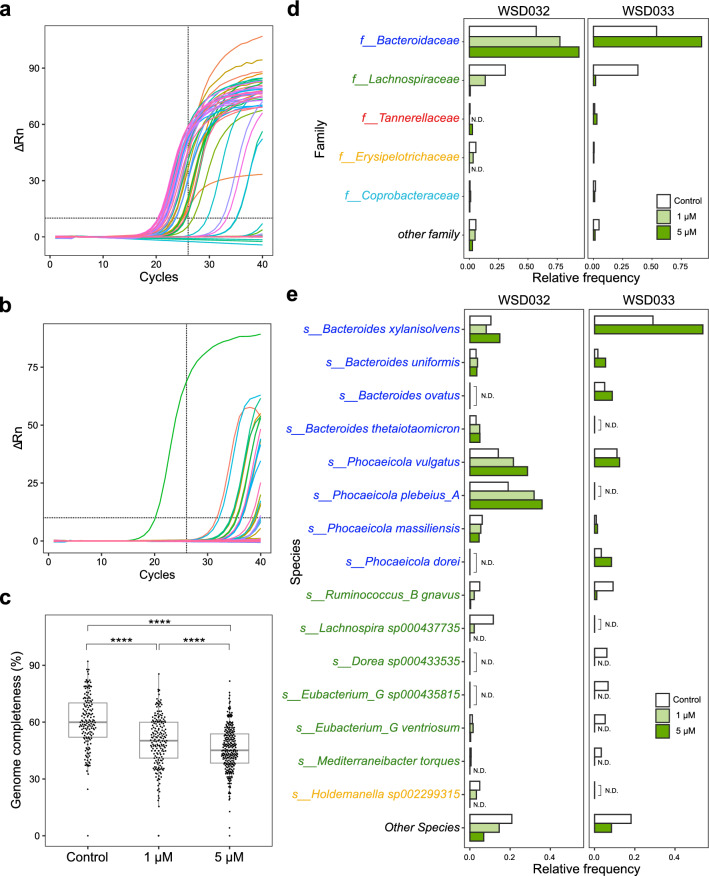
Single-cell sequencing of viable bacteria from human feces. (**a**, **b**) Detection of *E. coli* SAGs in spiked fecal samples via qPCR. Heat-killed *E. coli* cells were spiked into human feces-derived bacterial cells and then processed without (**a**) or with (**b**) PMA treatment, followed by SAG-gel. Crosshairs show thresholds for the positive amplification of *E. coli* DNA. All values were assessed for subject WSD033. (**c**) Genome completeness of human fecal bacteria SAGs obtained using PMA-SAG-gel with two PMA concentrations (1 μM and 5 μM). Control means no PMA treatment. ****p < 0.001 (Wilcoxon rank-sum test). All values were assessed for subject WSD032, and SAGs with < 1 Mb reads were removed. (**d**, **e**) Detected bacterial composition at the family level (**d**) and species level (**e**) in two fecal samples, WSD032 and WSD033, treated with PMA (N.D. not detected). The family and species names are colored according to family name. Other families or species accounted for less than 1.1% or 1.0%, respectively.

Since we confirmed that PMA could eliminate dead bacterial DNA from being amplified in a cultured bacteria model, we then evaluated human fecal samples, WSD032 and WSD033. First, to examine whether PMA can eliminate dead bacteria in the fecal bacterial community, heat-treated *E. coli* cells were spiked into a fresh fecal sample so that the *E. coli* present in fecal bacteria were 1/3 of the total number of cells. Single-cell genome amplification of *E. coli*-spiked fecal bacteria was conducted using PMA-SAG-gel. For each SAG, *E. coli*-specific qPCR primers were used to evaluate whether the SAG was of *E. coli* or of other fecal bacteria. As a result, 37 out of 94 SAGs (39.4%) without PMA were suggested to be *E. coli* SAGs (Fig. 4a). In the PMA-treated sample, only one out of 94 SAGs was an *E. coli* SAG (Fig. 4b). Sequencing was performed to determine whether this one *E.coli* SAG in the PMA-treated sample was derived from the culture strain or the host gut, and the similarity was evaluated through average nucleotide identity (ANI) analysis. The ANI between the extracted genome from the culture strain and the *E. coli* K12 reference genome was 99.9%, whereas the ANI between the qPCR-positive *E. coli* genome and the reference genome was 98.1% (Supplementary Table S1). This suggested that the *E. coli* present in the PMA-treated samples (Fig. 4b) was not derived from the cultured K12 strain. Taken together, the results indicated that PMA-SAG-gel can effectively eliminate dead bacteria present within the complex fecal bacterial community.

Next, we carried out whole-genome sequencing of viable multi-species bacteria in feces. Two PMA concentrations (1 μM and 5 μM) were used. The average size of the single-cell sequence data was less than 120 Mb. Total 1331 human fecal bacterial SAGs were obtained (175 SAGs (control), 192 SAGs (1 μM) and 275 SAGs (5 μM) from subject WSD032, and 384 SAGs (control) and 305 SAGs (5 μM) from subject WSD033). Since we adjusted the number of SAG acquisitions for each condition to allow for adequate data analysis, the differences in the number of SAGs and PMA concentration here do not explain the samples’ nature. The average genome completeness decreased from 56.1% in non-PMA-feces to 49.0% (1 μM) and 44.5% (5 μM) in PMA-feces (Fig. 4c). Moreover, the incidence of incomplete SAGs (completeness 0%) tended to decrease in PMA-feces (7.4% in non-PMA-feces, 2.1% in PMA(1 μM)-feces, and 3.3% in PMA(5 μM)-feces), indicating that PMA treatment eliminates free nucleic acids and may prevent the risk of their amplification along with SAGs.

Regarding the bacterial composition profile (Fig. 4d,e), *Lachnospiraceae* and *Erysipelotrichaeceae* accounted for more than 35% of bacteria in the non-PMA-feces of subject WSD032, while the SAGs of these bacteria were reduced in PMA-feces, with 90% being derived from *Bacteroidaceae* in 5 μM PMA-feces. The family *Bacteroidaceae* includes oxygen-tolerant commensal bacteria and is known to be enriched in PMA-treated feces^3,4^. In this study, fecal samples were collected under aerobic conditions, and our findings are consistent with previous results describing the recovery of genes derived from viable *Bacteroidaceae* members. A similar trend of variation in the species obtained following PMA treatment was also observed in another test subject (WSD033), with *Bacteroidaceae* as the main enriched family.

Since the fecal-derived bacteria (e.g., *Lachnospiraceae*) eliminated as dead bacteria in this study are mainly Gram-positive partial anaerobes, it may be possible to control their viability by maintaining appropriate collection and storage conditions. Thus, the currently described method may also be used to verify the optimal conditions for microbial survival in biological samples.

### Obtaining high-quality strain-resolved genomes by selective sequencing of *Bacteroidaceae*

**Figure 5:**
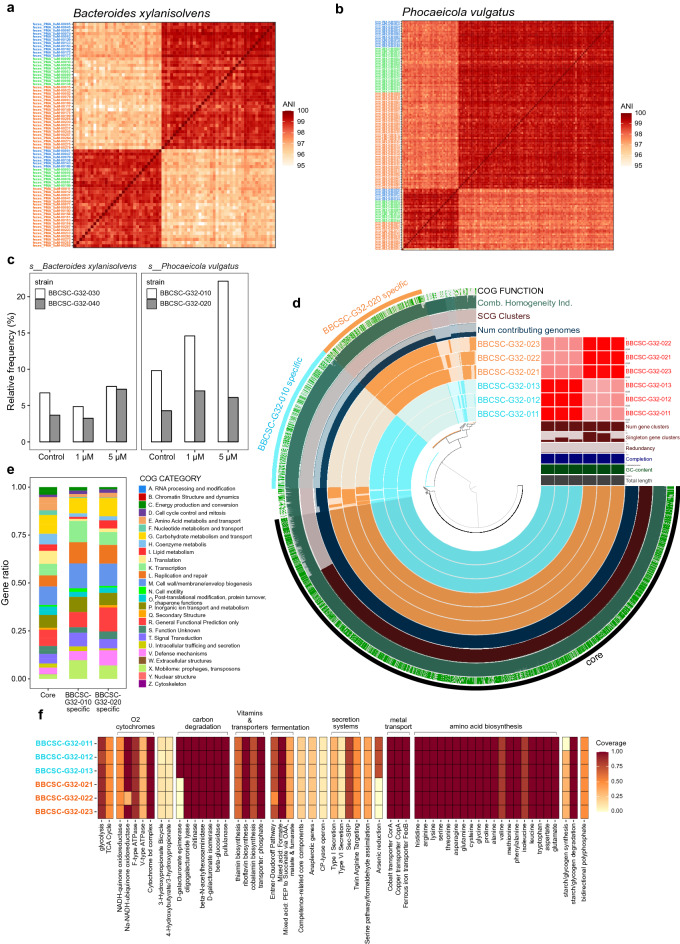
Strain-level genome analysis of *Bacteroidaceae* bacteria obtained from human feces. (**a**, **b**) Hierarchical clustering of pairwise average nucleotide identity (ANI) values across SAGs in *Bacteroides xylanisolvens* (**a**) and *Phocaeicola vulgatus* (**b**). SAGs for which ANI can be calculated for more than 75% of same-species SAGs, and the maximum ANI was more than 98.5%, were adopted as the data set. (**c**) Relative abundance of *B. xylanisolvens* (BBCSC-G32-030 and BBCSC-G32-040) and *P. vulgatus* (BBCSC-G32-010 and BBCSC-G32-020) strains as functions of PMA treatment concentration. (**d**) Anvi' o representation of *P. vulgatus* composite SAGs obtained from each PMA concentration. The first six layers represent each genome, and the blue/orange coloration indicates the existence of a gene cluster (GC). To the right is given an ANI percentage identity heatmap of the same genomes. The subsequent three layers correspond to various statistics related to the analysis, that is, the number of contributing genomes per GC, species core genome clusters, homogeneity index, and cluster of orthologous groups of proteins (COG) function. The outer green layer describes the gene groups in which at least one gene was functionally annotated using COGs. (**e**) COGs distributions in core and accessory genes of the two *P. vulgatus* strains. (**f**) KEGG Decoder heat map of the two *P. vulgatus* strains based on KEGG annotation. The heat map represents the metabolic pathway completeness of the SAGs based on the presence or absence of genes.

We focused on the enrichment of *Bacteroidaceae* from fecal samples through viable single-cell sequencing with PMA-SAG-gel. We obtained 245 *Bacteroidaceae* family SAGs out of 262 SAGs, including *Bacteroides thetaiotaomicron*, *Bacteroides uniformis*, *Bacteroides xylanisolvens*, *Phocaeicola massiliensis*, *Phocaeicola plebeius*, and *Phocaeicola vulgatus*. The composition of each species in the SAGs of subject WSD032 changed from 3.1 to 5.0%, 3.1 to 3.4%, 10.4 to 14.9%, 6.1 to 4.6%, 19.0 to 35.9%, and 14.1 to 28.6% after PMA treatment (Fig. 4e). Since more than six medium-quality draft genomes (completeness > 50%, contamination < 10%) were obtained, these ANIs were calculated (Fig. 5a,b), and composite SAGs were generated by merging the strain-specific SAG. As shown in Table 1, we obtained four *Bacteroides* spp. and four *Phocaeicola* spp. near-complete genomes. In particular, *Bacteroides xylanisolvens* (Fig. 5a) and *Phocaeicola vulgatus* (Fig. 5b) sequencing data clearly showed the presence of two different strains (BBCSC-G32-030 and BBCSC-G32-040 for *B. xylanisolvens*; BBCSC-G32-010 and BBCSC-G32-020 for *P. vulgatus*) within the same host, WSD032 (Fig. 5c). For *P. vulgatus*, PMA treatment resulted in the preferential enrichment of one strain (Fig. 5c). Comparison at the whole-genome level indicated that the two strains of *P. vulgatus* share many core genes, with specific accessory genes proprietary to each strain (Fig. 5d).

**Table 1:** Statistics of the composite single-amplified genome (SAG) of *Bacteroidaceae* family.

Species	PMA concentration	Completeness (%)	Contamination (%)	Total length (Mbp)	N50 (bp)	Number of contigs	GC content (%)	Number of CDS	n.5S	n.16S	n.23S	n.tRNA
*Bacteroides thetaiotaomicron*	0 μM	98.5	0.58	6.22	48,717	349	42.89	4908	1	1	1	17
	1 μM	97.3	0.43	6.19	57,339	288	42.88	4877	0	1	1	17
	5 μM	97.0	0.19	6.19	57,339	283	42.9	4870	0	1	1	16
*Bacteroides uniformis*	0 μM	98.9	0	4.92	63,695	209	45.87	4209	0	1	1	20
	1 μM	96.8	0.01	4.62	66,475	260	46.13	3902	0	1	1	19
	5 μM	97.3	0	4.73	67,399	222	46.24	3992	0	1	1	19
*Bacteroides xylanisolvens_* *BBCSC-G32-030*	0 μM	99.0	0	6.10	49,345	222	41.94	4765	1	1	1	19
	1 μM	99.0	1.18	6.10	44,101	310	41.93	4781	1	1	1	18
	5 μM	99.0	0	6.06	52,478	242	41.9	4729	1	1	1	18
*Bacteroides xylanisolvens_* *BBCSC-G32-040*	0 μM	97.9	0.39	6.27	46,497	359	41.8	5091	1	1	1	19
	1 μM	97.5	0.5	6.25	39,523	420	41.82	5058	1	1	1	18
	5 μM	99.3	0.07	6.27	48,493	288	41.77	5058	1	1	2	18
*Phocaeicola massiliensis*	0 μM	98.5	0.56	4.29	44,229	236	42.5	3738	1	1	1	17
	1 μM	98.9	0.56	4.28	44,229	207	42.56	3730	1	1	1	17
	5 μM	98.4	0.56	4.21	41,537	251	42.42	3649	1	1	1	19
*Phocaeicola plebeius_A*	0 μM	98.1	0.56	3.75	55,074	155	44.32	3021	1	1	1	14
	1 μM	97.0	0.74	3.71	59,262	127	44.23	2994	1	1	1	15
	5 μM	98.9	0.56	3.77	59,780	145	44.31	3033	1	1	1	13
*Phocaeicola vulgatus_* *BBCSC-G32-010*	0 μM	98.5	0	5.17	45,706	278	42.19	4376	2	1	1	18
	1 μM	98.1	0.38	5.20	48,149	265	42.23	4427	2	1	1	18
	5 μM	99.1	0	5.17	49,347	263	42.19	4376	2	1	1	18
*Phocaeicola vulgatus_* *BBCSC-G32-020*	0 μM	99.3	0.69	5.12	37,038	306	42.21	4318	1	1	1	17
	1 μM	98.6	0.69	5.12	36,217	312	42.19	4309	1	1	1	15
	5 μM	98.1	0.5	4.98	41,416	298	42.12	4160	1	1	1	17

Having identified two bacterial strains for *P. vulgatus* from a single host, we next examined their fundamental metabolic functions to identify strain-specific functions (Fig. 5e,f). The assessment of metabolic pathway completeness showed that strain BBCSC-G32-010 had high gene coverage of D-galacturonate epimerase and arsenic reduction pathway genes (Fig. 5f), while strain BBCSC-G32-020 lacked these. The Clusters of Orthologous Genes (COGs) distributions in the core and accessory genes are shown in Fig. 5e. The COGs in accessory genes showed different distributions to those in core genes and significantly enriched category L (Replication and repair) and category X (Mobilome: prophages, transposons) in both strains (Fisher’s extract test: p < 0.005). The characteristics of the accessory gene suggest that there are traits acquired by each strain to adapt to its environment or the incorporation of mobile genetic elements in the unique accessory gene of each strain. These results indicate that PMA-SAG-gel can be used to detect gene possession at the strain level and to gain insight into the physiological heterogeneity of bacterial strains.

## Discussion

While culture-based analyses have long been used to evaluate bacterial viability, many environmental microorganisms cannot be cultured. Therefore, culture-independent assessment of bacterial communities, especially through DNA sequencing, has been widely employed in recent years. However, culture-independent DNA analysis cannot clearly discern between viable and dead cells.

The lack of a method for evaluating bacterial community viability at the species or strain level necessitates the development of new approaches for specifically targeting the genomic content of living bacteria that are active within the environment^33–35^. PMA-coupled metagenomics has been employed for assessing the composition of viable bacteria. While this method has also been utilized to prevent misinterpretation of bacterial composition and activity due to dead bacteria in samples, metagenome-assembled genome (MAG) acquisition from uncultured viable microorganisms has not been well validated^36,37^. In particular, single-cell sequencing has been proposed to acquire SAGs from microorganisms active in the bacterial community in combination with BONCAT or Raman spectroscopy^33,35^, but PMA and single-cell sequencing combined with the acquisition of viable SAGs has not yet been reported.

Single-cell genomics is an approach that allows for the culture-independent whole-genome sequencing of microorganisms^38^. In contrast to metagenomics, single-cell genomics recovers genomic sequences from individual cells without the need for clonality of the bacterial population. Conventional single-cell genome sequencing involves single-cell preparations on plates via FACS, and, in many cases, nuclear staining with SYTO 9 is used. Therefore, in principle, it is possible to eliminate dead bacteria through live/dead fluorescence staining. However, plate-based single-cell genome amplification has many technical problems, such as the size selection of bacteria through FACS, insufficient lysis of Gram-positive bacteria due to simple alkaline lysis, and limited throughput. Therefore, although the presence of dead cells may compromise the accuracy of genome sequencing^19,39^, viability itself has not been of particular importance as cells are immediately lysed after isolation.

In the present study, we developed an SAG-gel which converts the small genomes of various bacterial cells into amplified genomes in uniformly shaped capsules floating within a single tube throughout the process. The gel matrix was used for cell lysis, washing, and amplified DNA purification. This matrix maintains the compartmentalized genome during cell lysis, washing, and WGA processes, preventing cross-contamination and facilitating the protection of the amplified DNA for long-term storage. These features solved the problems in conventional single-cell genomics, which allowed us to evaluate the effects of cell viability and viable bacterial sequencing using samples of PMA-treated bacteria.

The PMA-SAG-gel effectively suppressed the amplification of single-cell genomes from dead bacteria, resulting in a single-cell genome sequencing output exclusive for viable bacteria. PMA treatment also suppresses the amplification of free DNA and reduces the incidence of inappropriate SAG with 0% completeness, which occurs in a certain number of single-cell sequence outputs. A method to remove host DNA-derived sequences by preventing free DNA amplification through PMA treatment has been previously reported in metagenomic analysis^12^, and we considered that the same effect was achieved with the PMA-SAG-gel. Although we used fecal samples in the present study, the PMA-SAG-gel may be effective for analyzing samples with high human DNA contamination, such as saliva, and is thus expected to improve sequencing costs.

One of the challenges associated with the use of PMA is the need to validate appropriate processing conditions for the sample^40^. While individual validation is to be performed based on sample characteristics, our data from the analysis of feces revealed that the bacterial composition of acquired SAGs changes based on the PMA concentration. Our sample, which was prepared under aerobic conditions, showed high enrichment of the *Bacteroidaceae* family, which is oxygen-tolerant and has been described as particularly viable in FMT samples^3,4^. The enriched *Bacteroidaceae* genomes compromised of *Bacteroides* spp. and *Phocaeicola* spp., including multiple strains of *Phocaeicola vulgatus* and *Bacteroides xylanisovens*. In the case of MAG construction, it has been reported that genome binning of the *Bacteroides* genus from fecal samples is difficult even through long-read sequencing^41^. This difficulty arises from the fact that there is a large mixture of genomically similar organisms in the community, resulting in a consensus genome for the genus.

The PMA-SAG-gel enables the acquisition of genomes for a wide variety of *Bacteroides* bacteria and thus could collect as well as distinguish the genomes of different strains. Interestingly, in the sample from one subject, PMA-SAG-gel analysis revealed the changing of preferential survival in one *P. vulgatus* strain (BBCSC-G32-010) as functions of PMA treatment concentration. This finding confirmed that the two *P. vulgatus* strains detected in the current work had unique accessory genes. We also found the presence of arsenic resistance genes only in one of the two strains (BBCSC-G32-010). It is known that human gut bacteria, including *Bacteroides* sp. and *Phocaeicola* sp., may or may not harbor arsenic resistance genes, which varies between species or strains^42^. *P. vulgatus* is considered to have arsenic-responsive genes that confer resistance to inorganic arsenic and thus can persist in the gut following dietary exposure to inorganic arsenic^43^. Our results suggest that bacterial viability is different even within the same species, and we were able to identify specific functional differences through strain-resolved whole-genome analysis. The method described herein will further our understanding of the factors that enhance diversity and survival within the same species through the specific analysis of viable bacteria.

## Conclusions

In this study, PMA-SAG-gel enabled single-cell genome sequencing of surviving uncultured bacteria and identified specific viable bacterial strains in fecal samples processed under aerobic conditions. The technique generated massive SAGs covering various viable bacterial species, especially oxygen-tolerant *Bacteroidaceae*, and yielded high-quality draft genomes at the strain resolution. The strain-resolved analysis makes it possible to estimate accessory genes in each strain and the differences in metabolic functions or the presence of mobile genetic elements between the strains. In addition, because the output is in single-cell units, quantitative evaluation is possible. The survival profile of an entire population at the species or strain level will provide information for understanding the characteristics of the surviving bacteria under specific environments, sample processing, and insights for the quality assessment of live bacterial products and FMT as well as for understanding the acquisition of antibiotic resistance during treatment.

## Supplementary Information

Supplementary Table S1.

## Data Availability

Sequencing data have been deposited in the NCBI database under BioProject PRJNA776656. https://www.ncbi.nlm.nih.gov/bioproject/PRJNA776656.

## Abbreviations

SAG: Single-amplified genome
FMT: Fecal microbiota transplantation
PMA: Propidium monoazide
MDA: Multiple displacement amplification
GuSCN: Guanidine thiocyanate
COGs: Clusters of orthologous genes
